# COVID-19 Vaccine Anaphylaxis: Current Evidence and Future Approaches

**DOI:** 10.3389/falgy.2021.801322

**Published:** 2021-12-23

**Authors:** Wannada Laisuan

**Affiliations:** Division of Allergy Immunology and Rheumatology, Department of Medicine, Faculty of Medicine, Ramathibodi Hospital, Mahidol University, Bangkok, Thailand

**Keywords:** COVID-19 vaccine safety, anaphylaxis, IgE mediated, allergy, hypersensitivity

## Abstract

Vaccine anaphylaxis is rare; however, severe allergic reactions after administration of a coronavirus disease 2019 (COVID-19) vaccines have been reported. Excipients in the vaccine may play a role in severe allergic reactions post-vaccination. Various mechanisms, including IgE-mediated pathways, direct mass cell stimulation via the Mas-related G protein-coupled receptor-X2, and complement pathway activation, have been proposed to cause the anaphylaxis. Skin testing, using the basophil activation test, has been used to clarify the mechanism of the anaphylaxis and provide safety information for the next injection. Here, we review the current evidence and suggested approaches for patients who experienced an immediate severe allergic reaction to the first dose of a COVID-19 vaccine.

## Introduction

Owing to the outbreak of severe acute respiratory syndrome coronavirus 2 (SARS-CoV2), the US Food and Drug Administration (FDA) approved an emergency use authorization or two highly effective coronavirus disease 2019 (COVID-19) vaccines from Pfizer-BioNTech (1) and Moderna (2) on December 2020. A third vaccine, the Janssen COVID-19 vaccine, was authorized for use by the US FDA on February 27, 2021 (3). Following the implementation of COVID-19 vaccination programs, severe allergic reactions presenting as anaphylaxis have been reported.

Several criteria for anaphylaxis diagnosis have been proposed by the National Institutes of Allergy and Infectious Diseases (NIAID) (4) and the World Allergy Organization (WAO) (5). All organizations have accepted the concept that anaphylaxis is an “acute onset, serious, systemic, allergic, or hypersensitivity reaction that can be life-threatening or fatal” (6). Regarding immunization safety assessment, the Brighton Collaboration case definition criteria for anaphylaxis were developed in 2004 to facilitate the comparability of immunization safety data and distinguish different levels of diagnostic certainty (7).

According to NIAID in 2006 (4), anaphylaxis has rapid onset with more than one organ involved following an exposure to a likely allergen. However, a severe allergic reaction can present with only one organ system involved, such as isolated respiratory or cardiovascular symptoms (8). In addition, skin/mucosal involvement may be absent in 10–20% of all episodes, and decreased blood pressure in infants is often unrecognized (9), resulting in underreporting of anaphylaxis (10). Therefore, WAO in 2020 (5) amended the criteria for the diagnosis of anaphylaxis as acute onset of hypotension or bronchospasm or laryngeal involvement occurring in minutes to several hours after an exposure to a known or highly probable allergen, even in the absence of skin involvement.

Shimabukuro et al. have reported 21 cases of anaphylaxis according to the Brighton Collaboration case definition criteria for anaphylaxis after administration of 1,893,360 first doses of the Pfizer-BioNTech COVID-19 vaccine from December 14 to 23, 2020, corresponding to an estimated initial rate of 11.1 cases per million doses administered (11). Ten cases of anaphylaxis following 4,041,396 doses of the Moderna COVID-19 vaccine were reported in the Vaccine Adverse Event Reporting System (VAERS) from December 21, 2020 to January 10, 2021, representing 2.5 cases per million doses. From December 14, 2021 to January 18, 2021, 66 case reports met the Brighton Collaboration case definition criteria for anaphylaxis received by VAERS: 47 following administration of the Pfizer-BioNTech vaccine and 19 following the Moderna vaccine, corresponding to reporting rates of 4.7 and 2.5 cases per million doses administered, respectively (12). COVID-19 vaccine immunization can reduce the risk and severity of SARS-CoV2 infection (13). Therefore, understanding the mechanism of the allergic reaction and providing safety guidance for the next injection are crucial. This article reviews the causative mechanism, risk stratification, and the guidance for safety in anaphylaxis caused by COVID-19 vaccination.

## Allergic Reactions to COVID-19 Vaccines

Vaccine hypersensitivity reactions are infrequent, estimated to be 1.31 cases per million vaccine doses from a population-based study (14). Most reports of vaccine adverse reactions are not severe. However, severe allergic reactions, such as anaphylaxis, can occur (15). Immunological-mediated hypersensitivity can have either an immediate or a delayed onset of action (16).

**Figure 1:**
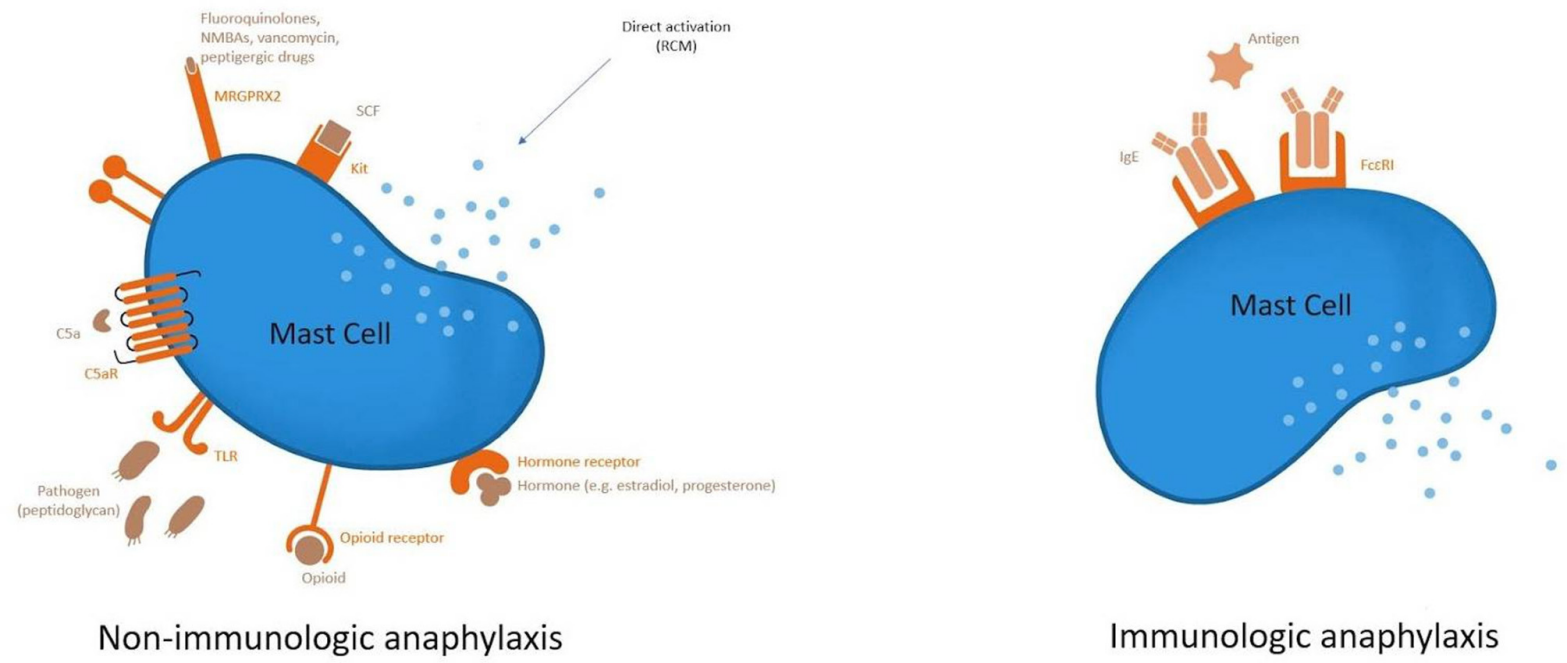
Mechanism of severe allergic reaction post-vaccination.

Type I hypersensitivity reactions can manifest as severe life-threatening anaphylaxis and typically occur in minutes or within 4 h of exposure. An IgE-mediated systemic reaction to the excipients in the COVID-19 vaccine may play a role in the potential causative mechanism of the anaphylaxis as in Figure 1. Systemic reactions involving multiple organs have resulted from the release of mediators caused by mast cell degranulation (17).

Complement activation-related pseudoallergy (CARPA) is another mechanism considered as a cause of the severe allergic reaction. Pre-existing anti-PEG IgM may trigger the classical complement pathway entailing anaphylatoxins (C3a, C5a) release that causes hypersensitivity reaction (18–21).

## Suspected Allergenic Components of the Vaccine

Allergic reactions following COVID-19 vaccination can result from vaccine components, including the active immunizing antigen, adjuvants, preservatives, stabilizers, antibiotics, cell culture materials, and leached packaging components (22). The vaccine antigen rarely, if ever, causes an allergic reaction and any reaction is more likely to be caused by excipients in the vaccine (15). The excipients in the COVID-19 vaccines and their functions are described in Table 1 (14).

**Table 1:** Excipients of COVID-19 vaccine and their function.

**Excipient**	**mRNA BNT162b2 Pfizer**	**mRNA-1273 Moderna**	**Janssen**
Active	Nucleoside-modified mRNA encoding the viral spike glycoprotein of SAR-CoV-2	Nucleoside-modified mRNA encoding the viral spike glycoprotein of SAR-CoV-2	Recombinant, replication-incompetent Ad26 vector, encoding a stabilized variant of the SARS-CoV-2 spike (S) protein
Lipid nanoparticles: stability and transport of mRNA PEGylated lipids	2**-[(Polyethylene glycol)-2000]**-N,N-ditetradecylacetamide (ALC-0159), 1,2-distearoyl-sn-glycero-3-phosphocholine (DSPC), ((4hydroxybutyl)azanediyl)bis (hexane-6,1-diyl)bis(2-hexyldecanoate) (ALC-0315), cholesterol	1,2-dimyristoyl-rac-glycero-3-methoxypolyethylene glycol-2000 **(PEG2000 DMG)**, 1,2-distearoyl-sn-glycero-3-phosphocholine (DSPC), lipid SM-102 (patented ionizable lipid), cholesterol	
Buffer: stability of lipid nanoparticles/radical oxidation inhibition	Potassium dihydrogen phosphate/disodium phosphate dihydrate	Tromethamol/tromethamol hydrochloride, acetic acid/sodium acetate trihydrate	
Other stabilizers: ionic strength, surfactant, metal-ion chelates	Potassium chloride, sodium chloride		**Polysorbate 80**, 2-hydroxypropyl-β-cyclodextrin, citric acid monohydrate, trisodium citrate dihydrate, sodium chloride, ethanol
Thermostabilization	Sucrose	Sucrose	

Polyethylene glycol (PEG) has been identified as the cause of severe allergic reactions (23–25). mRNA COVID-19 vaccines contain PEG2000 in the PEGylated lipids, which are used to form lipid nanoparticles that stabilize and transport the mRNA. Although PEG has not previously been used in a vaccine, PEG is used in compounds for polymer-based drug delivery and household products, such as cosmetics (26). Polyethylene glycol-associated anaphylaxis during colonoscopy preparation or laxative use has occurred in an average of four cases (range, 2–8) per year according to US FDA voluntary reporting data from 2005 to 2017 (23). Stone et al. have reported evidence for IgE-mediated hypersensitivity from skin and provocation testing in two patients with a history of anaphylaxis to PEG. One of the patients had skin test positivity and developed anaphylaxis symptoms during intradermal testing (23). However, non-IgE-mediated immune responses to PEG may be responsible for severe allergic reactions (19).

Polysorbate 80 is widely used as a stabilizing agent in food and pharmacological products, including vaccines. Previous studies have reported that polysorbate 80 can induce local and systemic allergic reactions, including IgE-mediated and non-immune anaphylaxis (27–29). Cross-reactivity to PEG has also been reported (23).

## Risk Stratification and Guidance for the Initial Administration of the COVID-19 Vaccine

According to the VAERS data from 55 states and US territories from December 14, 2020 to February 5, 2021, the relative incidence of anaphylaxis following administration of COVID-19 vaccines was two and seven times higher for recipients with a prior history of allergies and recipients who had experienced previous anaphylaxis, respectively (30). However, an allergy to food, drugs, inhalant allergens, or insect venoms is not a contraindication for vaccines. The only contraindications for vaccination with the COVID-19 vaccines are a proven diagnosis of vaccine component hypersensitivity or a previous history of severe allergic reaction to the first dose of the vaccine (31).

**Figure 2:**
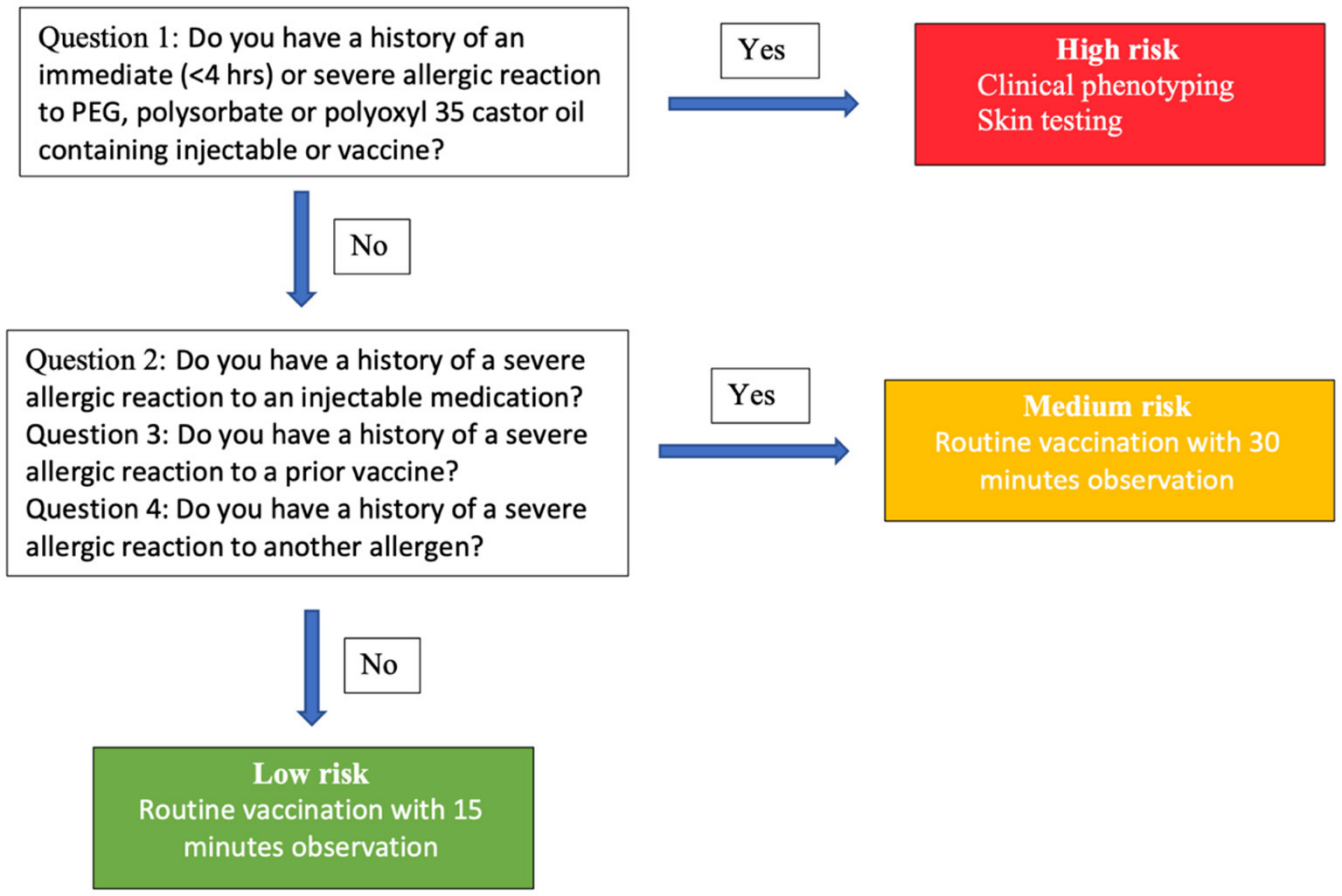
Risk stratification and guidance for initial administration of COVID-19 vaccine based on Mass General Brigham and Vanderbilt allergy expert consensus.

The Centers for Disease Control (CDC) has recommended pre-screening using a questionnaire for risk stratification as in Figure 2 (32). In individuals who have a history of an immediate (<4 h) or severe allergic reaction to a PEG-, polysorbate-, or polyoxyl 35 castor oil-containing injectable or vaccine, clinical phenotyping evaluation and skin testing with a standard concentration should be performed. In individuals who have a history of a severe allergic reaction to an injectable medication, a prior vaccine, or a history of a severe allergic reaction to another allergen, routine vaccination with observation for 30 min is recommended. If the answer to all the questions is “no,” the individual can receive a routine vaccination with 15 min observation.

## Diagnosis and Future Management of Vaccine Hypersensitivity

Skin tests are recommended for risk evaluation in patients with allergic reactions to COVID-19 vaccines by the WAO, American Academy of Allergy and Immunology (AAAAI), and European Academy of Allergy and Clinical Immunology (EAACI) but there is controversy over the recommendations for revaccination by graded administration (32–34). The Canadian Society of Allergy and Clinical immunology (CSACI) has suggested that skin testing is not required for a confirmed severe allergic reaction to a COVID-19 vaccine. However, graded administration can be considered in this case (35).

An individual who has a previous history of hypersensitivity to an excipient or has had a severe allergic reaction to the first dose of a COVID-19 vaccine should be evaluated by an allergist (36). Clinical phenotyping and shared decision making regarding the next injection should be performed. For an unconfirmed diagnosis who suspected allergic reaction to COVID-19 vaccine, a skin test to PEG and polysorbate at standard concentrations should be performed. However, the standardized concentration is not known (23).

Most patients who have had a previous severe allergic reaction to a COVID-19 vaccine can tolerate revaccination. Wolfson et al. have reported that 74% of patients who had an allergic reaction after the first dose of a mRNA vaccine and underwent allergic assessment by a skin test to PEG and polysorbate could be revaccinated safely. The negative predictive value of a negative skin test to PEG has been found to predict a 75% likelihood of tolerating the second dose of the vaccine (37). This study also found that patients who had a positive intradermal test to PEG (methylprednisolone acetate 4 mg/ml) could tolerate a second dose of mRNA vaccine without any reaction. Four patients who tested positive in a polysorbate skin test using refresh tears which contains polysorbate, received the second dose of mRNA vaccine safely, suggesting an irritant concentration of refresh tears (37).

Non-IgE-mediated allergic reactions may be responsible for COVID-19 allergies. Warren et al. performed allergy assessments by skin testing to PEG and polysorbate 80, basophil activation tests and measurement of PEG IgG levels in 11 patients who were suspected of having an allergic reaction to a mRNA COVID-19 vaccine (19). All the patients had positive basophil activation tests to PEG and mRNA vaccine; however, the skin tests to PEG and polysorbate 80 were negative.

The CDC have suggested an interchangeable vaccine platform using a vaccine that contains different excipients for patients who had reactions to excipients in the first dose of the vaccine. The adenoviral vector-based Janssen COVID-19 vaccine can be considered for individuals who had a severe allergic reaction to an mRNA-based COVID-19 vaccine. An mRNA COVID-19 vaccine can be considered for individuals who are contraindicated for the adenoviral vector-based Janssen COVID-19 vaccine. However, vaccination with caution is recommended because of the possibility of cross-reactivity between PEG and polysorbate 80 (38). Although graded challenge dosing has been used for other vaccines (39), there is no supporting data regarding the safety and efficacy of graded challenge dosing for mRNA vaccines.

Premedication with an antihistamine is controversial. The WAO (33), AAAAI (32), and BASCI (40) have recommended that premedication may mask the initial symptoms of a systemic reaction resulting in delayed diagnosis. However, pretreatment with a second-generation antihistamine can be considered in individuals with mild symptoms (pruritus or urticaria only) (32).

## Alternative Vaccine Options

Mixing and matching of two different COVID-19 vaccines could be an option if an individual is suspected to have had an allergic reaction to the first dose, or if there is a shortage of certain vaccine. Previous studies found priming with the ChadOx1 nCoV-19 vaccine followed by boosting with BNT162b2 resulted in significantly greater humoral and cellular immune responses (41–43). In solid organ transplant recipients who have limited humoral immune response to mRNA-based COVID-19 vaccines, heterologous vaccination seems promising because it induced specific antibodies and SARS-CoV2-specific CD4 and CD8 T cells (44).

Nordstrom et al. reported that individuals who used a heterologous ChadOx1 nCoV-19 and mRNA prime-boost vaccination strategy were 68% less likely to develop a symptomatic COVID-19 infection compared with those who were unvaccinated whereas those who used a homologous ChadOx1 nCoV-19 were 50% less likely to develop a symptomatic COVID-19 infection compared with those who were unvaccinated (45). Additionally, 88% effectiveness for preventing of SAR-CoV-2 infection was reported, which is similar to an individual receiving two doses of BNT162b2 (46).

Heterologous priming with the ChadOx1 nCoV-19 vaccine, followed by boosting with BNT162b2, is an effective alternative to induce strong humoral and cellular immune responses and acceptable reactogenicity (42, 46). This should be an alternative vaccination strategy for an individual who cannot complete the course using the same type of COVID-19 vaccine. However, there is a lack of evidence and knowledge about the necessity and timing of a booster dose following such a strategy.

## Conclusion

Allergic reactions to the COVID-19 vaccines are rare, and various components of the vaccines could be responsible for causing the reaction. To date, PEG and polysorbate 80 have been identified as possible factors for the etiology of reactions to the mRNA vaccine. However, the precise mechanism of action is not yet known. Therefore, the CDC has recommended that individuals with a history of anaphylaxis to PEG should avoid both COVID-19 mRNA vaccines. Furthermore, clinical phenotyping, allergic assessment, and shared decision making before the next injection should be performed in conjunction with an allergist. The future recommendations may change as additional data are obtained.

## Author Contributions

The author confirms being the sole contributor of this work and has approved it for publication.

## Conflict of Interest

The author declares that the research was conducted in the absence of any commercial or financial relationships that could be construed as a potential conflict of interest.

## Publisher's Note

All claims expressed in this article are solely those of the authors and do not necessarily represent those of their affiliated organizations, or those of the publisher, the editors and the reviewers. Any product that may be evaluated in this article, or claim that may be made by its manufacturer, is not guaranteed or endorsed by the publisher.
